# Posttraumatic cognitive appraisal, depression and quality of life among accidental trauma patients: a latent profile analysis and mediation analysis

**DOI:** 10.3389/fpsyg.2026.1961270

**Published:** 2026-10-19

**Authors:** Qianni Dong, Wenjing Yu, Baojian Wei, Wenyue Zhang, Aihua Zhang

**Affiliations:** 1 School of Nursing, Shandong First Medical University & Shandong Academy of Medical Sciences, Tai'an, Shandong, China; 2 Tai'an Municipal Hospital, Tai'an, Shandong, China; 3 Cancer Hospital of Shandong First Medical University, Jinan, Shandong, China

**Keywords:** accidental trauma, cognitive appraisal, depression, latent profile analysis, mediation, quality of life

## Abstract

**Background:**

Fostering positive cognitive appraisal among accidental trauma patients is critical for improving their quality of life. This study aimed to identify latent profiles of posttraumatic cognitive appraisal among accidental trauma patients, and to explore the associations among different cognitive appraisal profiles, depression and quality of life.

**Methods:**

A cross-sectional survey design was adopted. Assessment instruments included the Posttraumatic cognitive appraisal inventory (PTCAI), the Hospital anxiety and depression scale (HADS), and the Mos 36-item short form health survey (SF-36). Data in this study were analyzed using Mplus and SPSS. Latent profile analysis (LPA) was employed to identify the latent profiles of posttraumatic cognitive appraisal. Multinomial logistic regression was used to identify factors associated with the distinct posttraumatic cognitive appraisal profiles. One-way analysis of variance (ANOVA) was used to compare depression and quality of life across the distinct profiles. A mediation model with a multicategorical independent variable was used to examine the indirect associations between cognitive appraisal profiles and quality of life through depression.

**Results:**

A total of 459 accidental trauma patients were included. Three latent profiles of posttraumatic cognitive appraisal were identified: Negative cognitive appraisal group (14.6%), Neutral balanced cognitive appraisal group (56.2%), and Positive cognitive appraisal group (29.2%). Multinomial logistic regression analysis indicated that education, medical payment method, post-injury time, and injury severity score (ISS) were significant factors associated with different latent profiles (*p* < 0.05). Significant differences in depression and quality of life were observed across the three profiles. Taking the Neutral balanced cognitive appraisal group as the reference group, the indirect associations of depression were statistically significant for both pathways.

**Conclusion:**

Accidental trauma patients exhibit significant heterogeneity in posttraumatic cognitive appraisal. The indirect associations between cognitive appraisal profiles and quality of life through depression were statistically significant. Clinically, medical staff may consider stratified and individualized psychological interventions according to distinct cognitive appraisal profiles, which may help alleviate depressive symptoms and improve long-term quality of life.

## 1 Introduction

Accidental traumas, such as traffic crashes, falls and work-related injuries, are a major global public health problem (Chandran et al., 2010; Fogarty International Center National Institutes of Health, 2025). According to the World Health Organization (WHO), 4.4 million people worldwide die from injuries and violence each year. Of the 4.4 million injury-related deaths, unintentional injuries take the lives of 3.16 million people every year (World Health, 2024). Accidental traumas are sudden and unpredictable. They may not only cause physical injuries and functional impairments among survivors but also compromise mental health, manifesting as adverse affective outcomes including anxiety, depression, and persistent posttraumatic stress disorder (PTSD) (Wiseman et al., 2013; Connell and Olthuis, 2023; Namusoke et al., 2025). However, posttraumatic psychological responses vary considerably across individuals. While negative emotions emerge in the majority of individuals exposed to accidental trauma (Markovic et al., 2023; Rentmeesters and Hermans, 2023), a subset of people respond to traumatic events with positive optimism and experience positive psychological change (Doohan et al., 2018).

Cognitive appraisal, the evaluation of a stimulus or situation in terms of its meaning for the needs and goals of the individual (Lazarus and Folkman, 1984; Petri-Romão et al., 2025), functions as a key mediator of the link between trauma and stress responses (Kube et al., 2023; Gómez de La Cuesta et al., 2019). According to the cognitive appraisal theory proposed by Lazarus, psychological stress and emotional disorders are not directly induced by traumatic events themselves but are determined by individuals' subjective cognitive appraisal of trauma (Lazarus and Folkman, 1984). Sequential stages of cognitive appraisal are activated after accidental trauma. Primary appraisal centers on evaluating the threatening, loss-inducing and harmful attributes of trauma and determines the initiation of stress responses; secondary appraisal focuses on the assessment of available coping resources and adaptive capacities, which further modulates subsequent emotional adjustment (Folkman et al., 1986).

Cognitive theories of depression highlight that biased cognitive processes are critical to the development and maintenance of emotional disorders (Gotlib and Joormann, 2010; Beck, 2008). Negative cognition regarding traumatic loss, uncontrollable threats, and permanent physical and psychological impairment substantially increase the risk of adverse affective outcomes such as depression and hinder physical and mental recovery (Su and Liang, 2024; Mathews and MacLeod, 2005). In contrast, positive or adaptive cognitive appraisal serves a vital protective function in posttraumatic psychological recovery (Padmanabhanunni and Pretorius, 2023). Studies have demonstrated that posttraumatic positive cognitive appraisals, such as cognitive restructuring, benefit finding and posttraumatic growth, can effectively buffer the psychological impact of traumatic stress, reduce the cumulative effects of negative affect, facilitate the reconstruction of individual self-efficacy, and sustain stable mental functioning (Petri-Romão et al., 2025; Helgeson et al., 2006). Accordingly, posttraumatic cognitive appraisal is not a unidimensional negative processing pathway, but a multidimensional dynamic system coexisting with negative cognition and positive cognition. The combined pattern of these two cognitive components jointly shapes individuals' level of psychological adjustment (Zhang et al., 2023).

Quality of life serves as a comprehensive core indicator for evaluating rehabilitation prognosis and overall physical and mental health status among trauma patients (Meakes et al., 2024). Physical pain, functional limitations and altered life roles induced by accidental trauma directly disrupt patients' living conditions and lead to a marked decline in their overall quality of life (Afoakwah et al., 2025; Visser et al., 2021). In addition, existing studies have confirmed that depression is a prominent influencing factor of quality of life (Kosilek et al., 2026; Grauwmeijer et al., 2018). Accordingly, in-depth exploration of the intrinsic associations among profiles of cognitive appraisal, depression and quality of life in trauma patients carries important clinical implications for improving patients' long-term rehabilitation outcomes.

Most existing studies adopt variable-centered analytical approaches to examine linear associations between cognitive appraisal and psychological outcomes, while neglecting potential heterogeneity in cognitive patterns. As a person-centered statistical technique, Latent Profile Analysis (LPA) enables the identification of profiles with homogeneous comprehensive characteristics from heterogeneous populations, thereby revealing inherent heterogeneity among research participants (Howard and Hoffman, 2018). This method has been widely applied in mental health research and facilitates an in-depth understanding of the association between multiple factors and health outcomes.

Based on the above theoretical and empirical analyses, this study aims to reveal the latent profile of posttraumatic cognitive appraisal and its associated factors, as well as the differentiated pathways through which these distinct profiles are associated with patients' quality of life through depression. The findings may provide evidence for developing stratified and individualized psychological interventions to relieve depressive symptoms and improve patients' long-term quality of life.

The research hypotheses are as follows:

H1: accidental trauma patients exhibit distinct heterogeneous profiles of posttraumatic cognitive appraisal;

H2: accidental trauma patients across different latent profiles differ in depression and quality of life;

H3: cognitive appraisal is correlated with depression and quality of life, and the indirect associations between cognitive appraisal profiles and quality of life through depression are statistically significant.

## 2 Methods

### 2.1 Sampling and inclusion criteria

This study implemented a cross-sectional design and conformed to the Strengthening the Reporting of Observational Studies in Epidemiology (STROBE) reporting guidelines. Accidental trauma patients were recruited from the orthopedic and trauma departments of three tertiary-level hospitals in Shandong Province from January to June 2019. A convenience sampling method was used to recruit participants. All questionnaires were distributed and completed in paper form on site. Trained research personnel conducted face-to-face interviews with each participant, explaining the purpose and requirements of the survey and providing assistance throughout the questionnaire completion. Inclusion criteria: (1) patients had experienced an accidental trauma resulting in a physical impairment, defined as clinically documented structural or functional damage caused by the accidental trauma (e.g., fractures with limited joint range of motion, or sensory disturbances resulting from spinal cord or nerve injury); (2) patients were currently in the rehabilitation phase, defined as the period after the acute treatment phase (e.g., after surgical or conservative treatment) during which the participants' physical dysfunction or disability resulting from the accidental trauma was being addressed, mitigated, or eliminated through treatment and rehabilitation training; and (3) were aged 18 years or older. Exclusion criteria: (1) patients with communication disorders; (2) patients with a history of psychiatric disorders.

The sample size for this study was estimated based on Kendall's criterion (Lindley, 2005). Based on Kendall's method, the minimum sample size was estimated as 5-10 times the number of variables, and after accounting for a 10% invalid questionnaire rate, this yielded a minimum of approximately 356 participants. A minimum sample size of approximately 300 is recommended for LPA studies (Ferguson et al., 2020). A total of 490 questionnaires were distributed to eligible patients, and 459 valid questionnaires were returned, yielding an effective response rate of 93.7%.

### 2.2 Instruments

#### 2.2.1 Demographic characteristics

The instruments used in this study were designed by the research team following a literature review and preliminary studies (Zhang et al., 2023). The variables included in the final analysis were: gender, age, ethnicity, education, occupation, marital status, religion, medical payment method, cause of injury, history of intensive care unit, post-injury time, and injury severity. Objective injury severity was assessed using the Injury severity score (ISS) (Baker et al., 1974), which was dichotomized at the commonly used clinical cutoff of 16 for analysis (Boyd et al., 1987).

#### 2.2.2 The Posttraumatic cognitive appraisal inventory (PTCAI)

The Posttraumatic cognitive appraisal inventory (PTCAI) was developed by Zhang et al. and was tested for good reliability and validity (Zhang et al., 2023). It contains 27 items covering five dimensions: loss, feeling threatened, positively face, self-sense, and relationships. These five dimensions were further categorized into posttraumatic negative cognitive appraisal and positive cognitive appraisal. Scores for positive cognitive appraisal ranged from 16 to 80, with higher scores indicating higher levels of posttraumatic positive cognitive appraisal. Scores for negative cognitive appraisal ranged from 11 to 55, with higher scores indicating greater negative cognitive appraisal. In this study, the Cronbach's α coefficients for the negative and positive cognitive appraisal subscales were 0.824 and 0.859, respectively.

#### 2.2.3 The Hospital anxiety and depression scale (HADS)

The Hospital anxiety and depression scale (HADS) was developed by Zigmond and Snaith (Zigmond and Snaith, 1983). The HADS consists of 14 items, 7 of which evaluate depression and 7 of which evaluate anxiety. The depressive subscale of HADS was used in this study, with a score range of 0-21. The Cronbach's α for the depression subscale is 0.736.

#### 2.2.4 The Mos 36-Item short form health survey questionnaire (SF-36) - Chinese version

The Mos 36-Item short form health survey questionnaire (SF-36) was developed by Ware (Ware, 2000) to assess the quality of life, which was later translated into Chinese by Li et al. (2002). Eight dimensions of physical functioning, role physical, bodily pain, general health, vitality, social functioning, role emotional, and mental health, with 36 items, make up the two subscales of the SF-36 that measure physical and mental health, respectively. Scores vary from 0 to 100, with higher values reflecting higher quality of life. The Cronbach's α of the scale was 0.828.

### 2.3 Statistical analysis

All statistical analyses were performed using SPSS software (version 27.0), PROCESS macro (version 4.2), and Mplus software (version 8.3), with statistical significance set at a two-tailed *p* < 0.05.

Categorical variables were described using frequencies and percentages. Normality tests were performed for continuous variables. The skewness and the kurtosis of the core variables were less than |3| and |10| respectively (Table 1). According to Kline (Kline, 2011), the core variables in this study conform to an approximately normal distribution. Thus, continuous variables were expressed as mean and standard deviation (SD). Pearson correlation analysis was conducted to examine the relationships among cognitive appraisal, depression, and quality of life.

**Table 1:** Mean±SD and correlations among the major variables in this study.

Variable	Negative cognitive appraisal	Positive cognitive appraisal	Depression	Quality of life	Mean ±SD	Skewness	Kurtosis
**Negative cognitive appraisal**	1	-	-	-	30.50 ± 5.696	0.359	−0.364
**Positive cognitive appraisal**	−0.849^***^	1	-	-	62.18 ± 5.879	−0.734	0.488
**Depression**	0.494^***^	−0.489^***^	1	-	7.58 ± 2.287	−0.225	0.325
**Quality of life**	−0.485^***^	0.464^***^	−0.481^***^	1	50.45 ± 9.595	−0.254	−0.883

^***^p < 0.001.

To mitigate the influence of different measurement scales on the LPA model estimation, the five dimensions of PTCAI were z-standardized prior to analysis. Akaike Information Criterion (AIC), Bayesian Information Criterion (BIC), and sample-adjusted BIC (aBIC) were used to assess the fitting level of models. Lower information criterion scores show better model fits. An entropy value was employed to indicate the accuracy of the latent profiles, with the value closer to 1 indicating a more accurate classification. If the value exceeds 0.8, the accuracy of the latent profiles is greater than 90%. The likelihood ratio test consists of the Lo-Mendell-Rubin likelihood ratio test (LMRT) and the Bootstrap likelihood ratio test (BLRT). A significant LMRT or BLRT (*p* < 0.05) suggests that the k-class model fits the data better than the k-1 class model. After determining the optimal profile solution, chi-square tests and multinomial logistic regression were performed to examine differences in the distribution of demographic variables across cognitive appraisal profiles. To compare differences in depression and quality of life across these profiles, the ANOVA was used for overall comparisons. If the overall test was statistically significant, *post hoc* comparisons were conducted with the Bonferroni correction.

To explore the indirect role of depression between the different profiles and quality of life, a mediation model with a three-category independent variable was constructed. Given the cross-sectional design, the mediation analysis was used to examine indirect associations rather than to infer causal pathways. In this model, profiles derived from LPA (with Class 2 as the reference) were treated as the independent variable, depression as the mediator, and quality of life as the dependent variable. Independent-samples *t*-test or ANOVA were used to examine differences in quality of life scores across the baseline demographic and clinical characteristics, and variables showing statistically significant differences were then included as covariates (Table 2). The indirect association was tested using the Bootstrap method (5,000 resamples). A significant statistical association was considered present if the 95% confidence interval did not include zero. Statistical significance was set at a threshold of α = 0.05.

**Table 2:** Descriptive statistics of participants (*N* = 459).

Variables	Classification	*N*	%	t/F	*P*
**Gender**	male	289	63.0	−0.310	0.757
	female	170	37.0		
**Age (years)**	≤ 44	224	48.8	0.061	0.941
	45-59	176	38.3		
	≥60	59	12.9		
**Ethnicity**	Han	426	92.8	0.409	0.683
	other	33	7.2		
**Education**	middle school or below	210	45.8	8.849	**< 0.001**
	high school	132	28.8		
	junior college and above	117	25.5		
**Occupation**	worker	323	70.4	0.918	0.400
	self-employed	75	16.3		
	other (unemployed/retired)	61	13.3		
**Marital status**	married	373	81.3	1.626	0.105
	unmarried/divorced/widowed	86	18.7		
**Religion**	no	431	93.9	1.664	0.097
	yes	28	6.1		
**Medical payment method**	resident medical insurance	201	43.8	6.300	**0.002**
	employee medical insurance	160	34.9		
	self-paid	98	21.4		
**Cause of injury**	work-related injury	224	48.8	0.247	0.782
	traffic accident	189	41.2		
	other	46	10.0		
**History of intensive care unit**	yes	146	31.8	−3.152	**0.002**
	no	313	68.2		
**Post-injury time (months)**	≤ 3	220	47.9	13.500	**< 0.001**
	>3	239	52.1		
**Injury severity score (ISS)**	< 16	371	80.8	13.585	**< 0.001**
	≥16	88	19.2		

Bold values indicate *p* < 0.05.

## 3 Results

### 3.1 Common method bias

Harman's single-factor test was used to assess potential common method bias. The results revealed that 18 factors had eigenvalues greater than 1, and the first factor accounted for 18.01% of the total variance, which was below the critical threshold of 40%. These findings indicate that common method bias was unlikely to be a major concern.

### 3.2 Participant characteristics and preliminary analysis

The final sample comprised 459 accidental trauma patients. The majority of participants were male (*n* = 289, 63%) and under 45 years of age (*n* = 224, 48.8%). The vast majority were of Han ethnicity (*n* = 426, 92.8%). Most had an ISS of less than 16 (*n* = 371, 80.8%). Table 2 presents the remaining characteristics. Pearson correlation was used to examine the relationships among variables (Table 1). The results showed that negative cognitive appraisal was significantly positively correlated with depression (*r* = 0.494, *p* < 0.001), and negatively correlated with quality of life (*r* = −0.485, *p* < 0.001). Positive cognitive appraisal was significantly negatively correlated with depression (*r* = −0.489, *p* < 0.001) and positively correlated with quality of life (*r* = 0.464, *p* < 0.001). In addition, depression was significantly negatively correlated with quality of life (*r* = −0.481, *p* < 0.001).

### 3.3 Latent profile analysis

As shown in Table 3, the AIC, BIC, and aBIC values decreased monotonically as the number of latent profiles increased from one to five. Entropy values were above 0.80 for all models. The LMRT for the four-profile model was not statistically significant (*p* = 0.104), indicating that the four-profile model did not provide a significant improvement in fit over the three-profile model. Furthermore, the 5-profile model produced a class containing only 2.4% of the sample (*n* = 11), which lacked interpretability. Consequently, based on the combination of statistical fit indices, the principle of parsimony, and substantive clinical utility, the three-profile model was selected as the optimal solution. To verify the reliability of the results, we calculated the average posterior probabilities for each class and detailed data are presented in Supplementary Table 1.

**Table 3:** Fit indices for five models using latent profile analysis.

Profile	AIC	BIC	ABIC	Entropy	LMRT (*p*)	BLRT (*p*)	Proportion (%)
1	6527.922	6569.213	6537.476	-	-	-	-
2	5404.523	5470.588	5419.809	0.968	< 0.001	< 0.001	81.7/18.3
**3**	**4946.602**	**5037.441**	**4967.620**	**0.920**	**< 0.001**	**< 0.001**	**14.6/56.2/29.2**
4	4854.469	4970.083	4881.219	0.842	0.104	< 0.001	14.6/30.3/30.7/24.4
5	4779.370	4919.758	4811.852	0.900	0.011	< 0.001	28.5/14.6/22.7/31.8/2.4

*AIC, Akaike information criterion; BIC, Bayesian information criterion; ABIC, sample-adjusted BIC; LMRT, Lo-Mendell-Rubin likelihood ratio test; BLRT, Bootstrapped likelihood ratio test*.

Bold values indicate the selected optimal profile.

Figure 1 illustrates the standardized z-scores on the indicator variables for each of the three profiles, which represented 14.6% (*n* = 67), 56.2% (*n* = 258), and 29.2% (*n* = 134) of the sample, respectively. According to the differences in their relative levels and structural characteristics across these five dimensions, the profiles were named as follows: Negative cognitive appraisal group (Class 1), Neutral balanced cognitive appraisal group (Class 2), Positive cognitive appraisal group (Class 3).

**Figure 1:**
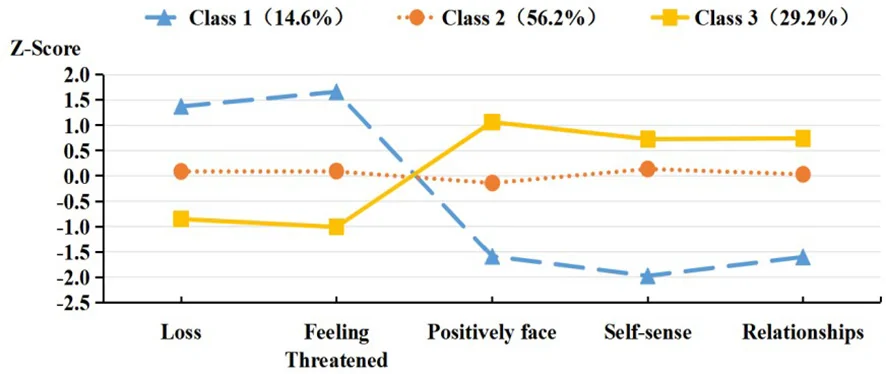
Latent profile characteristics of cognitive appraisal.

### 3.4 Differences in depression and quality of life across profiles

The ANOVA was used to compare depression and quality of life across the three profiles (Table 4). The results showed that there were significant differences in depression and quality of life (*p* < 0.001). The *post hoc* test revealed that the scores for depression in Class 1 were significantly higher than those of the participants in the other two classes (*p* < 0.001). In addition, Class 1 scored significantly lower than the other classes in terms of quality of life (*p* < 0.001).

**Table 4:** Differences in depression and quality of life across profiles.

Class	1	2	3	*F*	*Post hoc* tests
Depression	9.55 ± 1.861	7.76 ± 1.922	6.25 ± 2.312	59.507^***^	1>2>3
Quality of life	42.16 ± 7.652	49.91 ± 9.312	55.65 ± 7.617	70.640^***^	3>2>1

Class 1 = Negative cognitive appraisal group, Class 2 = Neutral balanced cognitive appraisal group, Class 3 = Positive cognitive appraisal group; ^***^p < 0.001.

### 3.5 Univariate and multinomial logistic regression analysis of factors associated with different cognitive appraisal profiles

Univariate analysis revealed statistically significant differences in education, medical payment method, history of intensive care unit, post-injury time and injury severity of the three profiles (Supplementary Table 2). Multinomial logistic regression analysis was performed with Class 1 as the reference group. The results showed that education, medical payment method, post-injury time and injury severity were identified as significant associated factors of cognitive appraisal profiles (Supplementary Table 3). In terms of education, participants with a middle school education or below were likely to fall into Class 1 or Class 2. Regarding medical payment methods, compared with self-payment, resident medical insurance and employee medical insurance were associated with a greater probability of being assigned to Class 2 or Class 3. The post-injury time of ≤ 3 months was linked to a higher chance of belonging to Class 3. Participants with an ISS below 16 were more likely to be classified into Class 2 or Class 3.

### 3.6 Indirect association of depression between different latent profiles and quality of life

To test the indirect association between cognitive appraisal profiles and quality of life through depression in accidental trauma patients, the cognitive appraisal profiles identified via LPA were entered into the model as independent variables, with Class 2 designated as the reference group. Education, medical payment method, history of intensive care unit, post-injury time, and ISS were included as control variables. As shown in Figure 2 and Table 5, all path coefficients in the model were statistically significant. Further analysis of the indirect effects showed that all 95% confidence intervals calculated by the bootstrap method did not include zero. Specifically, compared with the Neutral balanced cognitive appraisal group, the indirect effect of depression in the Negative cognitive appraisal group accounted for 42.46% of the total effect, and the indirect effect of depression in the Positive cognitive appraisal group accounted for 42.18% of the total effect (Table 6).

**Figure 2:**
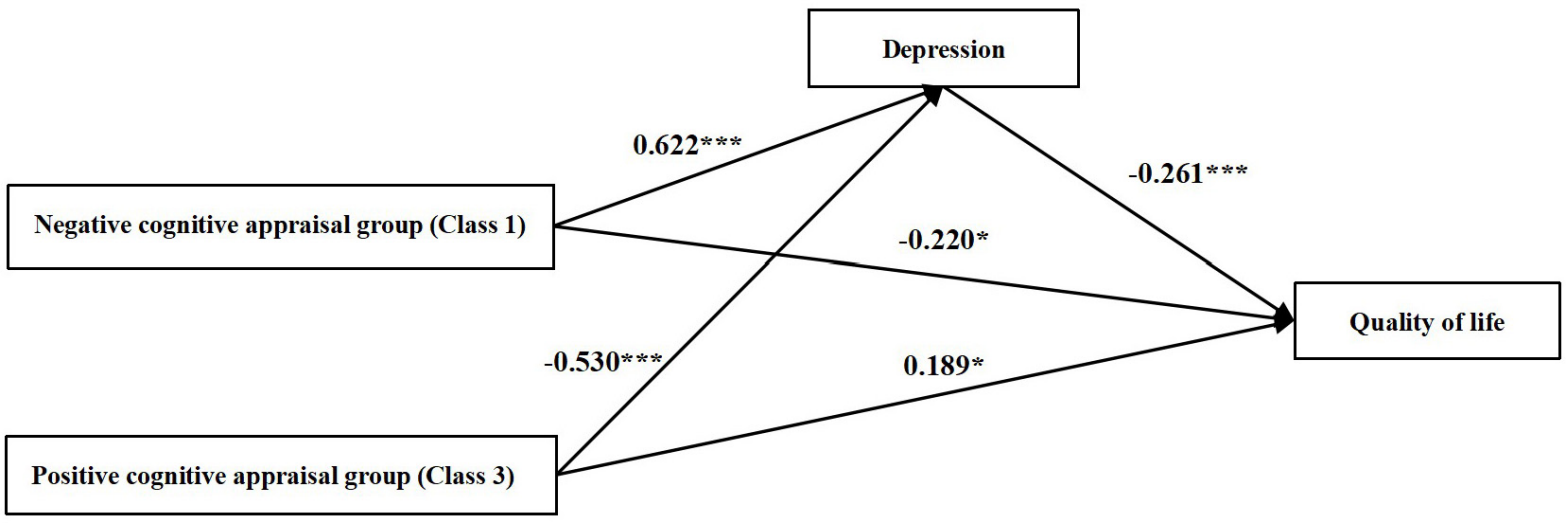
The indirect associations between cognitive appraisal profiles and quality of life through depression. ^*^p < 0.05, ^***^p < 0.001.

**Table 5:** Regression models for the mediation analysis of depression.

Regression equation		*R*	*R^2^*	*F*	β	*t*	*p*	95%CI
Outcome variables	Predictive variables							
Depression	Class 1	0.498	0.248	21.258	0.622	4.833	< 0.001	[0.369, 0.874]
	Class 3				−0.530	−5.389	< 0.001	[−0.723, −0.336]
Quality of life	Class 1	0.690	0.477	51.227	−0.220	−1.995	0.047	[−0.436, −0.003]
	Class 3				0.189	2.236	0.026	[0.023, 0.356]
	Depression				−0.261	−6.629	< 0.001	[−0.338, −0.183]

*Class 1* = *Negative cognitive appraisal group, Class 2* = *Neutral balanced cognitive appraisal group, Class 3* = *Positive cognitive appraisal group; R represents the multiple correlation coefficient and R*^2^ *represents the proportion of variance explained by the model*. β *values are standardized regression coefficients, and their significance was assessed by t-test. The 95%CI is the lower and upper limits of the 95% confidence intervals, respectively. Covariates including education, medical payment method, history of intensive care unit, post-injury time, and ISS*.

**Table 6:** Total, direct, and indirect effects in the mediation model.

Cognitive appraisal	Effect type	Effect	SE	LLCI	ULCI	Proportion (%)
**Class 1 (ref. Class 2)**	Total effect	−0.382	0.112	−0.603	−0.161	100.00%
	Direct effect	−0.220	0.110	−0.436	−0.003	57.57%
	Indirect effect	−0.162	0.040	−0.248	−0.088	42.46%
**Class 3 (ref. Class 2)**	Total effect	0.327	0.086	0.159	0.496	100.00%
	Direct effect	0.189	0.085	0.023	0.356	57.82%
	Indirect effect	0.138	0.033	0.078	0.210	42.18%

## 4 Discussion

Latent profile analysis identified three profiles of posttraumatic cognitive appraisal in this study: Negative cognitive appraisal group (Class 1), Neutral balanced cognitive appraisal group (Class 2), and Positive cognitive appraisal group (Class 3). We further explored factors associated with these profiles. Meanwhile, this study examined the indirect association between cognitive appraisal profiles and quality of life through depression.

### 4.1 Heterogeneity of posttraumatic cognitive appraisal among accidental trauma patients

Substantial heterogeneity exists in posttraumatic cognitive appraisal among patients with accidental trauma, and distinct patterns of cognitive appraisal can be observed across different individuals (Wang et al., 2022).

Class 1 accounted for the smallest proportion (14.6%). Patients in this class exhibited significantly higher scores on loss, feeling threatened and the total score of negative cognitive appraisal, whereas their total and each dimension scores of positive cognitive appraisal were markedly lower than those of the other two classes. Such patients tend to interpret traumatic events as irreversible losses and persistent threats. At the same time, they struggle to perceive the positive implications of their traumatic experience and are consequently susceptible to adverse emotional outcomes (Panagioti et al., 2012). From a clinical perspective, targeted cognitive restructuring interventions may be prioritized for this subgroup. Clinicians could guide patients to identify automatic negative thoughts about permanent functional impairment and uncontrollable trauma threats, then replace catastrophic interpretations with objective, balanced evaluations of their rehabilitation prospects. Meanwhile, regular brief psychological screening for depressive symptoms is recommended throughout their rehabilitation process to detect emotional distress at an early stage. Medical staff could also help patients recognize minor gains and positive changes during recovery, stepwise fostering their ability to discover beneficial meaning from traumatic experiences.

Class 2 represented the largest proportion (56.2%), constituting the predominant cognitive appraisal group among patients with accidental trauma. Scores across all cognitive appraisal dimensions of this class fell between Class 1 and Class 3, reflecting a balanced profile characterized by moderate negative and moderate positive cognition. This group may reflect the typical cognitive status of most individuals after trauma: they experience certain loss and threat perceptions, yet maintain a certain capacity for positive coping and social bonds without falling into extreme negative cognitive bias. Clinical interventions for this majority group could focus on consolidating their existing adaptive coping resources and preventing cognitive deterioration toward negative appraisal. Nurses and psychologists can deliver routine health education and regular emotional follow-ups to sustain their positive cognitive reserves and avoid the accumulation of trauma-related negative perceptions over prolonged rehabilitation.

Class 3 made up 29.2% of the sample. Patients within this class obtained the highest scores on positively face, self-sense, relationships and the total positive cognitive appraisal score, together with the lowest scores on negative cognitive appraisal dimensions. This reveals an adaptive cognitive pattern marked by low negative and high positive cognition. Individuals in this subgroup may possess greater psychological resilience and access to social support resources. They are capable of constructing positive meaning from traumatic experiences (Bonanno, 2004), which protects them against depressive emotions and sustains a relatively high quality of life. Clinical work for this population could mainly focus on maintaining their resilient cognitive strengths. Practitioners can guide them to share their positive coping experiences with other trauma patients to facilitate peer psychological support and amplify the protective effects of positive cognitive appraisal.

### 4.2 Associated factors of different profiles

The multinomial logistic regression analysis in this study identified education, medical payment method, post-injury time, and injury severity score as significant influencing factors of cognitive appraisal in patients with accidental trauma.

Firstly, in terms of education, patients with middle school education or below were significantly less likely to be classified into the Positive cognitive appraisal group compared to those with higher education, indicating that individuals with lower education levels tended to exhibit negative cognitive appraisal. A possible explanation is that individuals with lower education may lack effective emotion regulation strategies and are more prone to catastrophic thinking. In contrast, higher education may shape positive attitudes toward traumatic events by influencing health literacy, problem-solving skills, and channels of information acquisition (Zaman et al., 2024). Secondly, regarding medical payment method, patients with resident or employee medical insurance had higher odds of being classified into the Neutral balanced cognitive appraisal group or Positive cognitive appraisal group relative to self-paying counterparts. Under China's healthcare insurance framework, insurance coverage eases partial financial burdens arising from trauma treatment and relieves patients' anxiety over medical costs. Furthermore, medical insurance facilitates patients' access to higher-tier medical institutions (Wagstaff and Lindelow, 2008), enabling them to redirect cognitive resources from worrying about economic consequences toward active coping and rehabilitation. In contrast, self-paid patients face greater financial pressure, which may exacerbate negative appraisals of the traumatic event, such as perceived threat and loss of control. Notably, patients with a post-injury time of ≤ 3 months were more likely to be classified into the Positive cognitive appraisal group in this study, and this finding should be interpreted with caution. This phenomenon may be attributed to the high proportion of participants with mild injuries (ISS < 16) in our sample. Patients with minor injuries may achieve substantial recovery or even return to daily life within 3 months. Furthermore, optimistic bias (Harris and Hahn, 2011; Zoellner et al., 2008) or defensive denial (Dohrenwend et al., 2004) prevalent in the early posttrauma phase may lead patients to overestimate their positive cognitive appraisal shortly after the trauma. As time elapses, the reality of persistent functional impairment gradually emerges, and negative cognitive appraisal becomes more prominent. Future studies should adopt longitudinal designs combined with objective indicators to disentangle the independent effects of post-injury time and injury severity on posttraumatic cognitive appraisal. Finally, the injury severity score was an important factor associated with latent profile membership of cognitive appraisal. This finding is consistent with the results reported by Agar et al. (2006). Patients with ISS < 16 were significantly more likely to be classified into the Neutral balanced cognitive appraisal group or the Positive cognitive appraisal group. Patients with mild injuries generally face a lower threat of mortality, a shorter functional recovery period, and a reduced risk of permanent disability. These conditions enable individuals to maintain or rebuild a positive worldview and sense of self. In contrast, patients with severe injuries experience more intense threats to life, disruption of physical integrity, and a prolonged rehabilitation process, rendering them more vulnerable to persistent negative cognitive appraisal.

In summary, patients with higher educational attainment, medical insurance coverage, shorter post-injury time, and milder injuries in this study tended to be classified into the Neutral balanced or Positive cognitive appraisal groups. Therefore, individuals vulnerable to negative cognitive appraisal could be identified at an early stage and provided with cognitive restructuring interventions to facilitate their posttraumatic psychological adaptation. Meanwhile, multistage and continuous psychological support together with optimized medical policy guarantees could be delivered to patients requiring long-term rehabilitation treatment to alleviate their financial burden, thereby improving patients' cognitive appraisal and rehabilitation outcomes.

### 4.3 The relationships among cognitive appraisal, depression and quality of life

Significant differences were observed among the three profiles in terms of depression and quality of life. Class 1 exhibited significantly higher depression levels and lower quality of life scores than both Class 2 and Class 3. Indirect association analysis was further performed to examine the role of depression between cognitive appraisal and quality of life. Using the Neutral balanced cognitive appraisal group as the reference, both the Negative and the Positive cognitive appraisal groups showed significant indirect associations via depression. Specifically, negative cognitive appraisal was associated with elevated depression, and increased depression was in turn significantly related to reduced quality of life. This indirect pathway implies that negative cognitive appraisal may reinforce posttraumatic feelings of helplessness and negative automatic thoughts, and these cognitive appraisal processes are highly comorbid with depressive symptoms (Kleim et al., 2012; Majeed et al., 2021). Moreover, persistently high depression levels tend to be associated with poorer physical recovery, social functioning, and self-efficacy, which in turn may be linked to lower overall quality of life (Moergeli et al., 2012). Conversely, positive cognitive appraisal was linked to lower depression and better quality of life (Paquin et al., 2021; Riepenhausen et al., 2022; Richmond et al., 2014), suggesting that adaptive cognitive appraisal may facilitate the preservation or reconstruction of psychological resources. Therefore, for patients with neutral or positive cognitive appraisals, interventions may focus on consolidating existing adaptive cognitions to prevent the emergence of depressive symptoms. For trauma patients with negative cognitive appraisal, psychological interventions based on cognitive restructuring, such as cognitive reappraisal and cognitive behavioral therapy, could be delivered to alleviate negative emotions including depression, and thereby improve their long-term quality of life (Kam et al., 2024; Popiel et al., 2025).

### 4.4 Limitations and future directions

This study has several limitations. First, as a cross-sectional investigation, this study cannot establish causal or reverse relationships between variables. Longitudinal follow-up studies are needed to elucidate causal mechanisms. Second, the high proportion of participants with mild injuries (ISS < 16) may destabilize the association between post-injury time and cognitive appraisal patterns, so this result should be interpreted cautiously. Future studies may explore dynamic shifts in cognitive appraisal within different post trauma time frames. Third, most data were collected via self-report, which is susceptible to reporting bias. Subsequent research may integrate objective clinical indicators and observer-rated data. Lastly, the model did not account for all potential confounders. Therefore, future research with expanded covariate sets is necessary to further clarify the mediating pathways and to develop a more comprehensive analytical framework.

## 5 Conclusion

This study identified three heterogeneous profiles of posttraumatic cognitive appraisal among accidental trauma patients, with significant differences in depression and quality of life across the profiles. The indirect associations between cognitive appraisal profiles and quality of life through depression are statistically significant. These findings may offer potential implications for differentiated interventions aimed at improving cognitive appraisal.

## Data Availability

The raw data supporting the conclusions of this article will be made available by the authors, without undue reservation.
