# Supplementary material for: Posttraumatic cognitive appraisal, depression and quality of life among accidental trauma patients: a latent profile analysis and mediation analysis
Source: Front Psychol. 2026 Oct 19;17:1961270. doi: 10.3389/fpsyg.2026.1961270 (PMC13724429; doi:10.3389/fpsyg.2026.1961270)
Supplement: Supplementary file 1 [file Supplementary_file_1.docx]

Supplementary Material

**1 Supplementary Table S1.** Average attribution probabilities for each latent profile

| **Class** | **1** | **2** | **3** |
| --- | --- | --- | --- |
| **1** | 0.998 | 0.002 | 0.000 |
| **2** | 0.000 | 0.970 | 0.030 |
| **3** | 0.000 | 0.060 | 0.940 |

**2** **Supplementary Table S2.** Univariate analysis of factors associated with cognitive appraisal profiles

| **Variables** | **Classification** | **Class 1** | **Class 2** | **Class 3** | **χ²** | **P** |
| --- | --- | --- | --- | --- | --- | --- |
| **Gender** | male | 45(67.2) | 158(61.2) | 86(64.2) | 0.920 | 0.631 |
|  | female | 22(32.8) | 100(38.8) | 48(35.8) |  |  |
| **Age(years)** | ≤44 | 40(59.7) | 121(46.9) | 63(47.0) | 5.803 | 0.214 |
|  | 45-59 | 22(32.8) | 105(40.7) | 49(36.6) |  |  |
|  | ≥60 | 5(7.5) | 32(12.4) | 22(16.4) |  |  |
| **Ethnicity** | Han | 63(94.0) | 238(92.2) | 125(93.3) | 0.317 | 0.854 |
|  | other | 4(6.0) | 20(7.8) | 9(6.7) |  |  |
| **Education** | middle school or below | 39(58.2) | 133(51.6) | 38(28.4) | 35.399 | **<0.001** |
|  | high school | 21(31.3) | 71(27.5) | 40(29.9) |  |  |
|  | junior college and above | 7(10.4) | 54(20.9) | 56(41.8) |  |  |
| **Occupation** | worker | 44(65.7) | 174(67.4) | 105(78.4) | 6.469 | 0.167 |
|  | self-employed | 12(17.9) | 45(17.4) | 18(13.4) |  |  |
|  | other (unemployed/retired) | 11(16.4) | 39(15.1) | 11(8.2) |  |  |
| **Marital status** | married | 50(74.6) | 207(80.2) | 116(86.6) | 4.594 | 0.101 |
|  | unmarried/divorced/widowed | 17(25.4) | 51(19.8) | 18(13.4) |  |  |
| **Religion** | no | 62(92.5) | 240(93.0) | 129(96.3) | 1.876 | 0.391 |
|  | yes | 5(7.5) | 18(7.0) | 5(3.7) |  |  |
| **Medical payment method** | resident medical insurance | 17(25.4) | 120(46.5) | 64(47.8) | 20.602 | **<0.001** |
|  | employee medical insurance | 23(34.3) | 87(33.7) | 50(37.3) |  |  |
|  | self-paid | 27(40.3) | 51(19.8) | 20(14.9) |  |  |
| **Cause of injury** | work-related injury | 34(50.7) | 122(47.3) | 68(50.7) | 1.861 | 0.761 |
|  | traffic accident | 29(43.3) | 108(41.9) | 52(38.8) |  |  |
|  | other | 4(6.0) | 28(10.9) | 14(10.4) |  |  |
| **History of intensive care unit** | yes | 29(43.3) | 84(32.6) | 33(24.6) | 7.320 | **0.026** |
|  | no | 38(56.7) | 174(67.4) | 101(75.4) |  |  |
| **Post-injury time (months)** | ≤3 | 15(22.4) | 115(44.6) | 90(67.2) | 38.542 | **<0.001** |
|  | >3 | 52(77.6) | 143(55.4) | 44(32.8) |  |  |
| **Injury severity score (ISS)** | <16 | 32(47.8) | 211(81.8) | 128(95.5) | 66.098 | **<0.001** |
|  | ≥16 | 35(52.2) | 47(18.2) | 6(4.5) |  |  |

**3 Supplementary Table S3.** Multinomial logistic regression analysis

| **Variables** | **Class 2 vs. Class 1** | | **Class 3 vs. Class 1** | | **Class 2 vs. Class 3** | |
| --- | --- | --- | --- | --- | --- | --- |
|  | **OR(95%CI)** | ***p*** | **OR(95%CI)** | ***p*** | **OR(95%CI)** | ***p*** |
| **Education (ref:** junior college and above**)** | | | | | | |
| middle school or below | 0.924(0.343-2.494) | 0.876 | 0.235(0.080-0.686) | **0.008** | 3.938(2.137-7.256) | **<0.001** |
| high school | 0.876(0.305-2.518) | 0.806 | 0.461(0.150-1.424) | 0.179 | 1.899(1.000-3.607) | 0.050 |
| **Medical payment method (ref:** self-paid**)** | | | | | | |
| resident medical insurance | 4.480(2.010-9.987) | **<0.001** | 3.130(1.194-8.206) | **0.020** | 1.431(0.711-2.882) | 0.316 |
| employee medical insurance | 2.098(1.019-4.319) | **0.044** | 2.403(1.003-5.754) | **0.049** | 0.873(0.447-1.705) | 0.691 |
| **History of intensive care unit (ref: no)** | | | | | | |
| yes | 1.185(0.616-2.280) | 0.611 | 0.946(0.436-2.056) | 0.889 | 1.252(0.725-2.162) | 0.419 |
| **Post-injury time (months) (ref:** >3**)** | | | | | | |
| ≤3 | 1.841(0.934-3.630) | 0.078 | 3.916(1.850-8.287) | **<0.001** | 0.470(0.295-0.749) | **0.001** |
| **Injury severity score (ISS) (ref:** ≥16**)** | | | | | | |
| <16 | 4.881(2.521-9.450) | **<0.001** | 14.130(5.078-39.319) | **<0.001** | 0.345(0.138-0.865) | **0.023** |
